# Aging in Place in Frontier Wyoming: Experiences of Older Adults

**DOI:** 10.1093/geroni/igaf122.2820

**Published:** 2025-12-31

**Authors:** Chelsea Davis-Hearn, Jeff Clark, Bernard Steinman

**Affiliations:** University of Wyoming, Laramie, Wyoming, United States; State of Wyoming, Cheyenne, Wyoming, United States; University of Wyoming, Laramie, Wyoming, United States

## Abstract

Aging in place (AIP) is a relevant concern for older adults living in remote communities, where isolation and limited resources are distinct challenges that are often faced. Many older adults prefer to remain in their homes and communities because these are more than just places to live; they are filled with memories, connections, and a strong sense of belonging. However, the ability to age in place in extremely rural settings presents significant challenges such as limited access to healthcare, transportation, and social services. Understanding these factors is essential to developing strategies that promote autonomy, safety, and well-being for older adults living in frontier regions. This study focused on the perspectives and lived experiences of 20 older adults currently aging in place in Wyoming’s frontier communities, which are defined as less than 7 people per square mile. Using in-depth interviews, the study gained insights into the physical, social, and environmental factors that influence people’s ability to age in place as well as the strategies they utilize to cope with the challenges faced in these remote settings. Findings suggest that strong emotional attachments to the sense of home and community may serve as powerful motivators for people remaining in place despite potential difficulties. Involvement in community activities and support from family, friends, and neighbors proved essential for older people striving to maintain their independence. These findings inform the wider conversation on aging in place and reinforce the importance of social support systems for older adults residing in frontier areas.

